# Automatic Single-Cell Harvesting for Fetal Nucleated Red Blood Cell Isolation on a Self-Assemble Cell Array (SACA) Chip

**DOI:** 10.3390/mi15121515

**Published:** 2024-12-20

**Authors:** Hsin-Yu Yang, Che-Hsien Lin, Yi-Wen Hu, Chih-Hsuan Chien, Mu-Chi Huang, Chun-Hao Lai, Jen-Kuei Wu, Fan-Gang Tseng

**Affiliations:** 1 Department of Engineering and System Science, National Tsing Hua University, Hsinchu 30013, Taiwan; 2 Nano Science and Technology Program, Taiwan International Graduate Program, Academia Sinica and National Tsing Hua University, Taipei 11529, Taiwan; 3 Biomedical Science and Engineering Center, National Tsing Hua University, Hsinchu 30013, Taiwan; 4 Institute of Nano Engineering and Micro Systems (NEMS), National Tsing Hua University, Hsinchu 30013, Taiwan; 5 Frontier Research Center on Fundamental and Applied Sciences of Matters, National Tsing Hua University, Hsinchu 30013, Taiwan

**Keywords:** FnRBC, single-cell isolation, STR, NIPT

## Abstract

(1) Background: Fetal chromosomal examination is a critical component of modern prenatal testing. Traditionally, maternal serum biomarkers such as free β-human chorionic gonadotropin (Free β-HCG) and pregnancy-associated plasma protein A (PAPPA) have been employed for screening, achieving a detection rate of approximately 90% for fetuses with Down syndrome, albeit with a false positive rate of 5%. While amniocentesis remains the gold standard for the prenatal diagnosis of chromosomal abnormalities, including Down syndrome and Edwards syndrome, its invasive nature carries a significant risk of complications, such as infection, preterm labor, or miscarriage, occurring at a rate of 7 per 1000 procedures. Beyond Down syndrome and Edwards syndrome, other chromosomal abnormalities, such as trisomy of chromosomes 9, 16, or Barr bodies, pose additional diagnostic challenges. Non-invasive prenatal testing (NIPT) has emerged as a powerful alternative for fetal genetic screening by leveraging maternal blood sampling. However, due to the extremely low abundance of fetal cells in maternal circulation, NIPT based on fetal cells faces substantial technical challenges. (2) Methods: Fetal nucleated red blood cells (FnRBCs) were first identified in maternal circulation in a landmark study published in *The Lancet* in 1959. Due to their fetal origin and presence in maternal peripheral blood, FnRBCs represent an ideal target for non-invasive prenatal testing (NIPT). In this study, we introduce a novel self-assembled cell array (SACA) chip system, a microfluidic-based platform designed to efficiently settle and align cells into a monolayer at the chip’s base within five minutes using lateral flow dynamics and gravity. This system is integrated with a fully automated, multi-channel fluorescence scanning module, enabling the real-time imaging and molecular profiling of fetal cells through fluorescence-tagged antibodies. By employing a combination of Hoechst+/CD71+/HbF+/CD45− markers, the platform achieves the precise enrichment and isolation of FnRBCs at the single-cell level from maternal peripheral blood. (3) Results: The SACA chip system effectively reduces the displacement of non-target cells by 31.2%, achieving a single-cell capture accuracy of 97.85%. This isolation and enrichment system for single cells is well suited for subsequent genetic analysis. Furthermore, the platform achieves a high purity of isolated cells, overcoming the concentration detection limit of short tandem repeat (STR) analysis, demonstrating its capability for reliable non-invasive prenatal testing. (4) Conclusions: This study demonstrates that the SACA chip, combined with an automated image positioning system, can efficiently isolate single fetal nucleated red blood cells (FnRBCs) from 50 million PBMCs in 2 mL of maternal blood, completing STR analysis within 120 min. With higher purification efficiency compared to existing NIPT methods, this platform shows great promise for prenatal diagnostics and potential applications in other clinical fields.

## 1. Introduction

The increasing maternal age in many parts of the world has significantly elevated the demand for prenatal examinations, which have become a cornerstone of maternal–fetal medicine. Over the past decade, technological advancements have revolutionized prenatal diagnostics, enabling earlier and more accurate evaluations of fetal health. Among these, serum-based biomarkers such as pregnancy-associated plasma protein-A (PAPP-A), first introduced in 1993, have served as predictors of pregnancy complications [1]. When combined with Free β-HCG, these markers achieved detection rates for trisomy 21 of approximately 90%, with false positive rates between 2% and 3%, depending on gestational age [2]. However, the scope of detectable conditions using these biomarkers remained narrow, paving the way for the development of non-invasive prenatal testing (NIPT) using cell-free DNA (cfDNA).

CfDNA-based NIPT involves the massively parallel sequencing of DNA fragments extracted from maternal plasma, allowing for the detection of fetal chromosomal abnormalities and small (>5 Mb) sub-chromosomal copy number changes. The abundance of cfDNA in maternal circulation and its relatively straightforward extraction process are notable advantages, making it one of the most widely used methods in clinical practice. However, cfDNA also has inherent limitations. Its short half-life (<2 h) and low fetal fraction (5–20%) can lead to false negative results. Additionally, cfDNA-based NIPT is considered a screening test, necessitating confirmation through invasive diagnostic methods [3]. While cfDNA demonstrates high sensitivity and specificity for detecting Down syndrome, it has slightly reduced sensitivity for Edwards and Patau syndromes. Moreover, the short fragment size of cfDNA restricts its utility in detecting a broader spectrum of genetic abnormalities [4].

An alternative to cfDNA for NIPT is found in fetal nucleated red blood cells (FnRBCs), which were first identified in maternal circulation in 1959 (*The Lancet*) and successfully isolated in 1990 (PNAS) [5]. FnRBCs have intact nuclei, offering a more comprehensive genetic blueprint compared to fragmented cfDNA. Early studies reported detection rates of 41.4% for fetal gender determination and 74.4% for chromosomal aneuploidy, with false positive rates ranging from 0.6% to 11.1% [6]. Despite its potential, FnRBC-based NIPT has faced challenges due to the rarity of these cells in maternal blood and technical difficulties in isolating them with sufficient purity for reliable analysis.

Cell sorting methods for FnRBCs can be categorized into fluorescence marker-based techniques [4,5], microbead capture [6,7,8], and marker-free physical sorting [9,10]. Fluorescence-based methods use immunofluorescent antibodies to label target cells, often in conjunction with flow cytometry, which enables sorting based on signal detection. Microbead capture methods employ antibody-coated beads, including magnetic beads, to selectively isolate FnRBCs. Marker-free physical sorting leverages intrinsic cell properties such as size, deformability, and density to distinguish and isolate specific cell populations [11]. Each of these approaches has strengths but also limitations, such as low throughput, time inefficiency, and high operational costs. Additionally, issues like contamination risks, difficulty in maintaining sterility, and large equipment size pose barriers to the widespread adoption of these methods in clinical settings.

Efforts to improve the precision, speed, and efficiency of cell sorting platforms have resulted in new technologies capable of addressing these challenges. Modern platforms aim to enhance throughput, reduce contamination risks, and streamline operations, while ensuring high purity and minimal cell loss. For example, advancements in automated imaging systems integrated with cell sorting mechanisms have significantly improved the performance of NIPT workflows. These systems not only enhance sorting purity but also minimize human error and reduce aerosol exposure, improving safety in laboratory settings. Despite these innovations, the low abundance of FnRBCs in maternal circulation remains a significant barrier to the broader application of cell-based NIPT.

During pregnancy, maternal erythrocytes may express CD71 (transferrin receptor) under specific physiological and pathological conditions, which can interfere with FnRBC isolation. For instance, increased erythropoiesis in pregnancy-related anemia elevates the proportion of reticulocytes that exhibit higher levels of CD71. Similarly, inflammatory conditions like preeclampsia disrupt erythropoiesis and lead to CD71 expression in maternal erythrocytes [12]. Additionally, fetal–maternal hemorrhage introduces fetal erythrocytes with naturally high CD71 expression into maternal circulation, complicating the differentiation of FnRBCs from maternal cells [13]. To address these challenges, combining specific markers such as HbF, CD71, and CD CD45− has been proposed to enhance the specificity of FnRBC isolation [14].

Moreover, selecting appropriate fluorescence-labeled antibodies is critical for improving the accuracy of FnRBC detection. CD147, for example, has been suggested as a potential marker for FnRBCs due to its expression in fetal cells [8,15]. However, its expression in other maternal blood components, including erythrocytes, leukocytes (e.g., lymphocytes and monocytes), extracellular vesicles, and endothelial cells, can lead to background signal interference, necessitating the careful optimization of antibody selection [16,17].

This study aims to advance the use of FnRBCs as a reliable target for NIPT by integrating a novel self-assembled cell array (SACA) chip with an automated imaging and sorting system. Our approach leverages a combination of HbF+, CD71+, and CD45− markers to achieve the high-purity isolation of FnRBCs, overcoming the limitations of traditional sorting methods. By utilizing this advanced microfluidic platform, we propose a robust and efficient solution for non-invasive prenatal diagnostics, offering the potential for more comprehensive genetic analysis and improved clinical outcomes.

## 2. Materials and Methods

### 2.1. Materials

Lymphoprep™ (PG-1114547-1) was obtained from Blossom Biotechnologies Inc., Taipei, Taiwan. ε-Polylysine was purchased from the JNC Corporation, Tokyo, Japan. PE Mouse Anti-Human Fetal Hemoglobin (560041) was supplied by BD Pharmingen through the UNIMED Corp., Taipei, Taiwan. Human TRA-1-85/CD147 Alexa Fluor^®^ 350-conjugated Antibody (FAB3195U) and APC Anti-Human CD45 Antibody (AF18395) were acquired from the TAQKEY Corp., Taipei, Taiwan. FITC Anti-CD71 (ARG62923) was sourced from the Arigo Biolaboratories Corp., Taipei, Taiwan. Hoechst dye was purchased from ABP Biosciences, LLC., Taipei, Taiwan.

### 2.2. Blood Sample Pretreatment

The blood samples were processed according to a density gradient centrifugation protocol. First, a 2 mL blood sample was centrifuged horizontally at 800× *g* for 15 min, at 4 °C to separate the peripheral blood mononuclear cells (PBMC). The mononuclear cells containing FnRBCs were transferred into a 15 mL centrifuge tube and centrifuged horizontally at 300× *g* for 10 min. The supernatant was removed and PBS solution of 100 μL total volume was added. The process flow is shown in Figure S1.

### 2.3. FnRBC Immunofluorescent Staining

Immunofluorescent staining was performed to identify FnRBCs. After blood sample pretreatment, the cells were incubated in immunofluorescent staining solution containing 20 μL PE-conjugated antibody HbF (0.2% *w*/*v*), 20 μL APC-conjugated antibody CD45 (0.2% *w*/*v*), and 20 μL of FITC-conjugated antibody CD71 (0.2% *w*/*v*) in the dark at room temperature for 30 min. The cells were nucleus-stained with Hoechst (0.1 mg/mL in deionized water) for 10 min and then washed with deionized water.

### 2.4. ε-Polylysine Coating

The SACA chip was treated with O_2_ plasma for 5 min, followed by the addition of 20 µL of ε-polylysine into its wells. The system was left undisturbed for 10 min and subsequently baked in an oven at 60 °C for 2 h.

### 2.5. FnRBC Screening, Isolation, and Release

After staining the cells, all cells were loaded into the SACA chip and incubate at room temperature for 5 min until the cells settled [18]. The automated cell imaging and sorting system began to perform three types of fluorescent and visible light scans. The operator performs FnRBC identification, location, and counting based on the three types of fluorescent image signals (Hoechst+/CD71+/HbF+/CD45−) after scanning and the cell types are observed by bright field. The cell isolation device moves to the FnRBCs’ position according to the coordinates selected by the user and is fitted with a high-precision micron-level syringe pump for the selection and storage of cells in the corresponding container for subsequent detection and analysis. The complete process flow is shown in Figure S1.

### 2.6. Whole Genome Amplification (WGA)

Whole genome amplification (WGA) was performed using the QIAGEN REPLI-G Kit (Qiagen Inc., Redwood City, CA, USA) following the manufacturer’s protocol with slight modifications. The sorted target cells were transferred into a PCR tube and centrifuged to collect the cell pellet. After centrifugation, the supernatant was carefully removed, leaving approximately 4 µL of liquid in the tube. Subsequently, 3 µL of Buffer D2 was added to the sample, mixed thoroughly, and incubated on a heating block at 65 °C for 10 min to facilitate cell lysis. After incubation, 3 µL of stop solution was added to the reaction, and the sample was placed on ice to halt the lysis process. Next, 40 µL of a master mix, consisting of an amplification buffer and polymerase, was added to the sample and mixed thoroughly to initiate the WGA reaction. The sample was incubated at 30 °C for 8 h on a heating block to allow for DNA amplification. Following the amplification step, the reaction was terminated by incubating the sample at 65 °C for 3 min. After the WGA process, the amplified DNA was analyzed for quality and concentration. This was achieved by measuring the DNA concentration and absorbance values (OD260/280) using an enzyme-linked immunosorbent assay (ELISA) reader. These steps ensured the accuracy and efficiency of the DNA amplification process for subsequent analyses.

### 2.7. Short Tandem Repeats (STR)

This experiment was conducted by the Genelabs Life Science Corp., New Taipei City, Taiwan, using their specialized testing services. The short tandem repeat (STR) analysis was performed using the Promega GenePrint^®^ 24 System kit, and the raw data was analyzed with GeneMapper^®^ Software V3.7. DNA samples were extracted and prepared following standard protocols to ensure sufficient concentration and purity for analysis. PCR amplification was performed using the Promega GenePrint^®^ 24 System kit specified reaction mixture and thermal cycling conditions to amplify the 24 STR loci. The amplified products were then subjected to capillary electrophoresis to separate STR fragments by size, following the manufacturer’s guidelines. The resulting raw electrophoresis data were imported into GeneMapper^®^ Software V3.7, where the STR alleles for each locus were identified and sized. Positive and negative controls were included to ensure accuracy and reliability. After the verification of the allele calls and consistency across loci, the STR profiles were compiled and documented for downstream applications, such as fetal–maternal comparisons or genetic analysis. All procedures were conducted under strict quality control to maintain the integrity of the results.

## 3. Results

### 3.1. Cell Isolation Device

The self-assembled cell array (SACA) chip, previously developed and published by our team [19], is an innovative microfluidic platform designed for the efficient enrichment and immunolabeling of rare cells from biological samples. By leveraging gravity and lateral flow dynamics, the SACA chip enables the rapid formation of a monolayer of cells without requiring external fluid control equipment, significantly simplifying the cell detection process. The resulting single-cell layer is illustrated in Figure S2.

**Figure 1:**
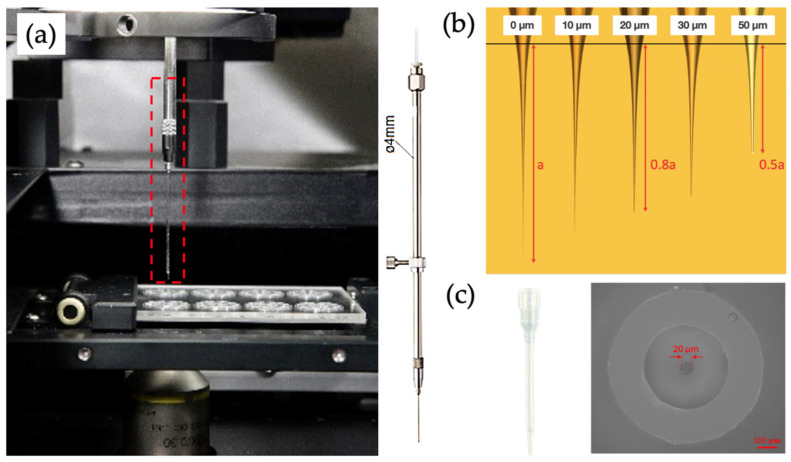
Cell isolation device. (**a**) The view inside the automatic cell image scanning and isolation system. The red cube represents the cell needle holder and the cell needle. (**b**) The relationship between the length of the needle tip and the inner diameter of the drawn glass capillary tube. (**c**) Structure of the plastic needle enclosing the glass needle and the microscopic field of view. Scale bar: 100 µm.

The cell sorting device comprises a high-precision syringe pump (SP101 was purchased by Yotec Instruments Co., Ltd., Taipei, Taiwan), a three-axis motor (Micromanipulator-MP285 was purchased by the Sutter Instrument Company, Novato, CA, USA), a cell needle holder, and a cell needle (Figure 1a). The syringe pump precisely controls the flow rate and volume during cell picking and releasing, which can be adjusted based on the number of target cells. For single-cell isolation, the flow rate is typically set at approximately 40 µL/min, while higher rates are required for cell clusters. The three-axis motor, with a movement accuracy of 1 µm, ensures the precise positioning of the target cell. The cell needle, fabricated from a glass capillary tube, features inner and outer diameters determined by the length of the truncated needle tip, ranging from a minimum inner diameter of 7 µm to a maximum of 50 µm (Figure 1b).

To integrate the newly developed automated image recognition software, we have further enhanced the system. The upgraded design incorporates a plastic needle to prevent damage to the chip while stabilizing the lateral and vertical positioning of the glass needle. This modification reduces the needle diameter from 400 µm to 20 µm—a 20-fold reduction—making it ideal for isolating target cells (FnRBCs) with diameters of approximately 12–18 µm, as shown in Figure 1c.

**Figure 2:**
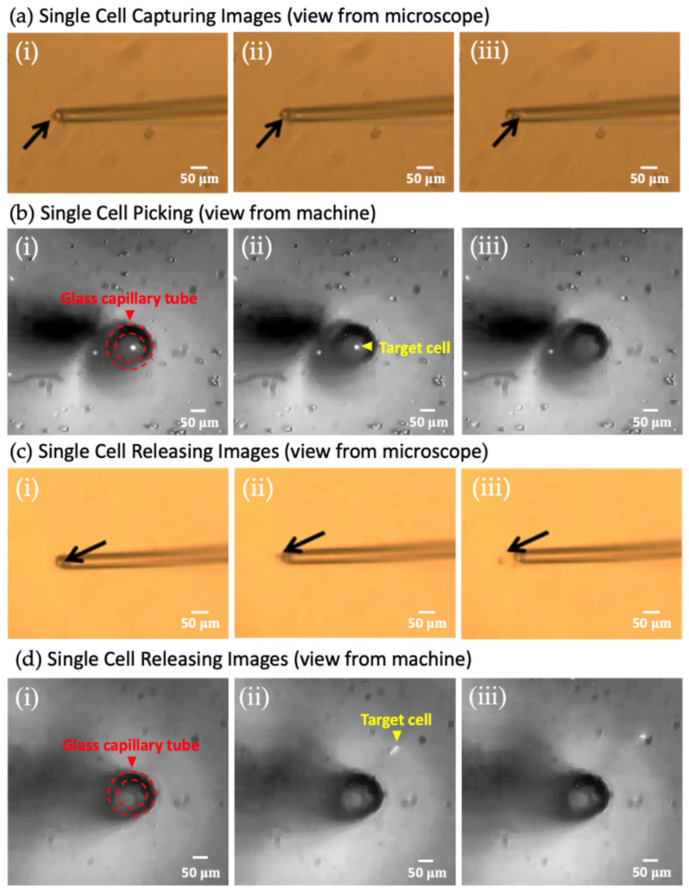
The process of single-cell isolation. (**a**,**b**) The process of single-cell picking. (**c**,**d**) The process of single-cell releasing. (i), (ii), and (iii) represent the processes of cells being aspirated or released, respectively. In Figures (**a**,**c**), the black arrows indicate the positions of cell movement within the microscopic field of view, while Figures (**b**,**d**) depict the top-down view of cell movement within the device.

The system first identifies the target cell’s position and uses a high-precision three-axis motor to maneuver the cell isolation device to the designated location. The device then employs precise control of the volume and flow rate to accurately aspirate the cell and release it into the corresponding storage container. The entire process of cell uptake and release is illustrated in Figure 2. This sequence of images demonstrates the isolation of a single FnRBC from a specific well of the SACA chip and its subsequent transfer to an empty well, ensuring precise and contamination-free manipulation.

In order to improve the accuracy of identifying FnRBCs, we incorporated ε-Polylysine (ε-PL) molecules synthesized by the JNC Corporation into our device. The ε-PL demonstrates a unique capability of forming NH_2_ functional groups upon binding to polycarbonate (PC) surfaces, imparting a positive charge to the surface. Given that cells exhibit a net negative charge in phosphate-buffered saline (PBS), the positively charged NH_2_ functional groups on the modified PC surface facilitate effective cell adhesion. As illustrated in Figure S3a, this modification allows for the efficient immobilization of cells on the chip surface, enabling straightforward retrieval by gently tapping the cells with a micropipette. As shown in Figure S3b, compared to the previously used F127, ε-PL coating effectively immobilizes most cells. Additionally, it reduces the number of non-target cells captured, resulting in a 31.2% decrease in the number of displaced cells during the cell-capturing process.

### 3.2. Precision of Single-Cell Isolation

**Figure 3:**
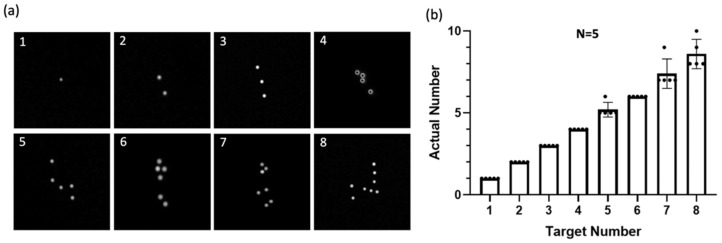
Precision test of single-cell isolation using the SACA system. (**a**) Representative images showing isolated cells from different target number groups (1–8 cells). (**b**) Statistical analysis of the number of FnRBCs isolated, repeated five times for each target group. The results demonstrate high precision, with isolated cell numbers closely matching the target numbers across all groups (*n* = 5). Error bars indicate the standard deviation.

To evaluate the sorting capability of the device, we conducted a precision quantification test for single-cell sorting. We specified the target cell number (1–8 cells) of the well of the SACA chip and isolated the cells from the well containing immunofluorescence-stained cells until we achieved the number of the target cell. After sorting, we performed automatic image scanning to calculate the actual number of cells in the well. The accuracy is evaluated through the relative error (Er), which is defined as Er = (Xi − Xt)/Xt × 100%, where Xi is the actual measured value and Xt is the target value. Figure 3a is the result after we have isolated the target cells in the new well, and Figure 3b is the statistical result of the number of specified target cells we have isolated five times. It can be seen from the graph that when the number of sorted cells is 1–4, the accuracy is 100%. When the sorting number is between 5–8 cells, there is a difference of ±2 cells between the actual number and the target number. According to the results of the experiments and calculations, the average relative error is about 2.15%, which means that this system has a high cell isolation ability with a sorting accuracy of 97.85%.

In addition, to evaluate the detection limit of FnRBCs on the automated system, we conducted an experiment to simulate conditions for sorting rare target cells, enabling potential application for pregnant samples at earlier gestational stages. Specifically, 20 FITC-labeled white blood cells were mixed into a background of 500 million unlabeled white blood cells (Figure S4a) and subjected to sorting. As shown in Figure S4b, the system successfully identified and isolated all 20 fluorescently labeled white blood cells, demonstrating its capability to detect rare targets even in complex cellular environments. This result establishes the system’s limit of detection (LOD) at 1 in 10^8, providing a robust foundation for future applications in NIPT.

### 3.3. FnRBCs Isolation

The immunofluorescence-stained cells are loaded into the well of the SACA chip, and each well can contain around 500,000 cells. The micro-structure and hydrophilic surface treatment drive the lateral flow field and the gravity field. The cells are flattened into a dense monolayer within five minutes. The single layer and tight cell arrangement effectively avoids cell stacking that can cause difficulty in image recognition or the sorting of non-target cells. After the cells have settled, four types of fluorescent and visible light automated image scanning are performed. FnRBCs can be identified by the cell types presented by fluorescent signals (Heochst+/CD71+/HbF+/CD45−) and visible light images.

**Figure 4:**
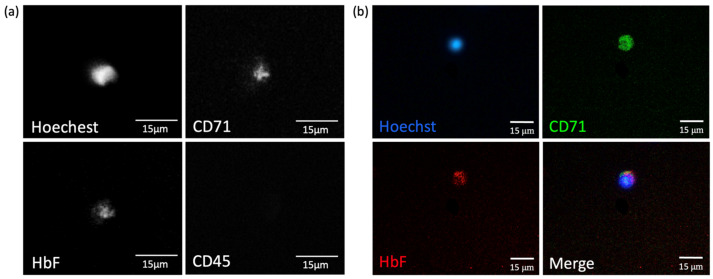
Images of FnRBCs on the SACA chip. (**a**) Signals (Heochst+/CD71+/HbF+/CD45−) from the cell imaging scanner before pickup. (**b**) Released FnRBC signals (Heochst+/CD71+/HbF+/CD45−) from a fluorescence microscope.

In addition to recognizing the FnRBCs’ signal from our automated machine, we also used a fluorescent inverted microscope for further confirmation. The FnRBCs’ signal from the automated cell imaging scanner (Figure 4a) is the same as the signal from the fluorescence microscope (Figure 4b), which is Heochst+/CD71+/HbF+/CD45−. Therefore, the cell an FnRBC. The sorted FnRBCs should be stored in the corresponding container for subsequent analysis, such as STR and other tests, for the verification of FnRBCs.

Additionally, we obtained blood samples from non-pregnant women and performed staining with different combinations of antibodies: (A) CD147+CD71+HbF+CD45−, (B) Hoechst+CD147+CD71+CD45−, and (C) Hoechst+CD71+HbF+CD45−, as shown in Figure S5. Statistical analysis revealed that the *p*-values for group B and group C compared to group A were 0.021079 and 0.03709, respectively. The results indicate that group B showed no significant difference compared to group A, suggesting that CD147 is less specific compared to HbF. Furthermore, the similarity between group B and group A highlights that the addition of CD147 does not provide significant advantages. Thus, HbF alone is sufficient for reliable identification. Based on these findings, we selected HbF and CD71 as the primary antibodies in our final staining protocol.

### 3.4. The Volume and the Background Noise of Single-Cell Isolation

Cell sorting is a critical step for downstream applications such as short tandem repeat (STR) analysis, which imposes an upper limit on the volume of the sorted sample. When a single cell is stored in a container with an excessive liquid volume, it can generate significant noise during subsequent cell signal amplification, complicating detection and analysis. Therefore, achieving cell sorting in an extremely small volume is essential to improve sorting purity and analytical precision.

**Figure 5:**
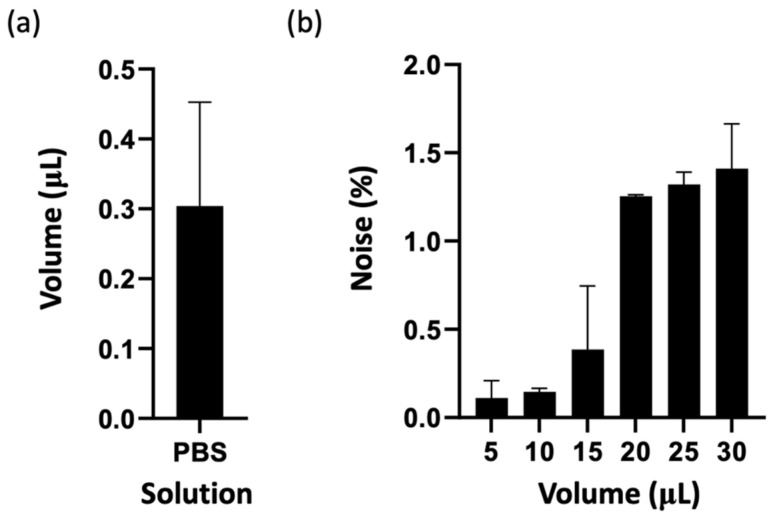
The average volume and background noise of single-cell isolation using PBS. (**a**) The average isolation volume for single cells is approximately 0.304 μL, demonstrating precise volume control during isolation (*n* = 3). Error bars indicate the standard deviation. (**b**) Background noise concentration at varying isolation volumes. Noise levels increase with larger isolation volumes, ranging from 0.11% at 5 μL to approximately 1.5% at 30 μL, highlighting the importance of minimizing isolation volume to reduce background interference (*n* = 3). Error bars indicate the standard deviation.

To address this, we conducted 20 single-cell sorting experiments and measured the volume of each using a contact angle microdroplet volume measurement technique. The results showed that the average volume of a single cell sorted by our device is approximately 0.304 μL, as illustrated in Figure 5a. This volume is approximately 1/100 of the upper detection volume limit for STR analysis, demonstrating its suitability for downstream applications. Using this average volume as a parameter, we configured the high-precision syringe pump for optimized single-cell sorting. While only a single cell is sorted, maternal free DNA in the solution may still be inadvertently transferred into the storage container during aspiration and release. To quantify the potential impact of background contamination, we used PBS containing uniformly distributed 0.37 μm fluorescent polystyrene (PS) beads at a defined concentration (100% background noise) and introduced the solution into the SACA chip wells. Six different sorting volumes (5, 10, 15, 20, 25, and 30 μL) were tested, and the absorbance values were measured using an ELISA reader (GloMax^®^ Explorer Multimode Microplate Reader, Promega Corporation, Madison, WI, USA). The calibration curve was used to calculate the corresponding background noise concentrations, as shown in Figure 5b.

The results indicated that the background noise for a 5 μL sorting volume was approximately 0.11%, with a positive correlation observed between sorting volume and background noise concentration. At a sorting volume of 20 μL, the background noise concentration approached saturation, and at 30 μL, the maximum background noise reached around 1.41%. However, given the average sorting volume of 0.304 μL in our single-cell sorting experiments, the background noise concentration is estimated to be less than 0.11%, specifically around 0.0067%, based on proportional volume percentage calculations.

This remarkably low sorting volume significantly minimizes background contamination, ensuring high sorting purity. Based on the results of these quantitative experiments, achieving a lower single-cell sorting volume not only complies with the upper volume limits required for subsequent analysis but also effectively reduces background noise, enhancing the reliability and accuracy of downstream applications.

### 3.5. Maternal Blood Sample for STR Test

To validate the potential application of our approach in NIPT, we designed an STR experiment to assess the accuracy of FnRBC isolation from maternal blood. Using the SACA chip and fluorescent antibody staining with Hoechst+CD71+HbF+CD45−, we isolated FnRBCs and evaluated their suitability for downstream DNA analysis.

Five groups of samples (labeled A to E) were subjected to whole genome amplification (WGA) following cell capture. The number of FnRBCs captured from each group was eight, five, three, three, and three, respectively, as shown in Figure S6a. The OD260/OD280 ratios of the extracted DNA ranged between 1.5 and 1.7, slightly below the ideal range of 1.7 to 1.9, indicating suboptimal purity. This deviation suggests minor protein or contaminant residues in the samples, likely due to the limited DNA quantity obtained from microvolume samples. Nevertheless, the observed purity is within an acceptable range for amplified microcell samples, demonstrating the feasibility of using the SACA chip and our antibody combination for isolating FnRBCs suitable for genetic analysis.

**Figure 6:**
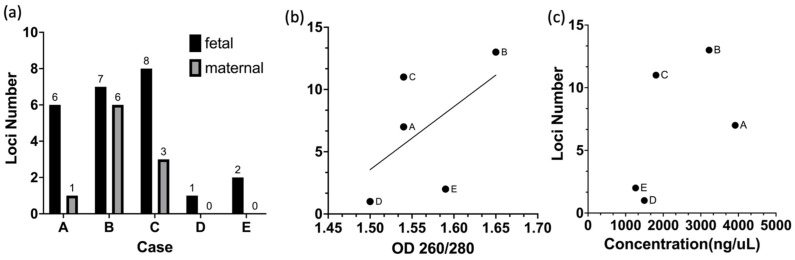
The STR results for FnRBCs isolated using the SACA chip and a Hoechst+CD71+HbF+CD45− antibody combination. (**a**) Comparison of detected loci numbers in fetal and maternal cells across five sample groups (A–E). (**b**) Correlation between OD260/280 ratios and the number of detected loci. (**c**) Relationship between DNA concentration and loci number.

As shown in Figure S6b, a positive correlation was observed between the number of cells and DNA concentration, with fewer cells yielding lower DNA concentrations. Furthermore, Figure 6a illustrates that our SACA system successfully captured fetal cells from maternal blood. In these results, ‘fetal’ refers to alleles where one copy originates from the father and the other from the mother, while ‘maternal’ refers to alleles identical to those of the mother. Across 24 loci, the fetal cells exhibited a higher number of signals compared to maternal cells. Figure 6b demonstrates a positive correlation between DNA quality and loci number, and Figure 6c shows that higher DNA concentrations further enhance the detection of additional loci.

These results emphasize the importance of capturing an adequate number of FnRBCs and maintaining high DNA quality to ensure sufficient DNA yield for downstream analysis. High-quality DNA contributes to the improved detection of genetic markers, supporting the reliability and accuracy of NIPT. This study provides a foundation for further optimization to enhance DNA quality and ensure robust downstream applications in NIPT.

## 4. Discussion

The precision and reliability of single-cell isolation demonstrated by the SACA chip, automated imaging, and sorting system are exceptional. With an average sorting accuracy of 97.85% and a relative error of approximately 2.15%, the system provides a reliable platform for isolating target cells, including rare populations such as FnRBCs. This high degree of precision is crucial for ensuring the purity and integrity of isolated cells, which is paramount for downstream analyses such as STR testing. Compared to conventional methods that often suffer from lower throughput and increased contamination risk, this technology represents a significant advancement in the field of single-cell sorting [20,21].

Although significant efforts have been made by various research groups to isolate circulating fetal cells (CFCs), particularly FnRBCs, and apply the technology in clinical settings, very few have reported successful results [22,23]. Many existing methods, such as fluorescence-activated cell sorting (FACS) and magnetic-activated cell sorting (MACS), involve labor-intensive and time-consuming experimental steps, limiting their practicality in clinical cytogenetics laboratories. In contrast, the SACA system achieves near automation in both cell capture and recovery processes, significantly reducing manual intervention and improving workflow efficiency. Our SACA system integrates a high-precision syringe pump, a three-axis motor, and an automated imaging scanning function, enabling the automatic localization and separation of FnRBCs. Its level of automation reduces the need for manual operations, minimizing errors caused by human intervention and enhancing the accuracy and efficiency of cell capture and recovery.

The system’s low detection limits and background noise reduction further highlight its efficacy. The detection limit (LOD) of 1 in 10^8, established by spike-in experiments using FITC-labeled white blood cells, underscores the platform’s ability to detect and isolate rare target cells even in highly complex biological samples. Additionally, the low average sorting volume of 0.304 μL minimizes background noise to less than 0.11%, making it suitable for sensitive analyses such as STR. These findings demonstrate that the platform can provide clean, high-quality cell samples, essential for applications like non-invasive prenatal testing (NIPT) [24].

The successful isolation of FnRBCs from maternal blood using Hoechst+/CD71+/HbF+/CD45− antibodies and subsequent STR analysis validates the FnRBC isolation efficiency and clinical feasibility of the SACA system. The positive correlation between DNA concentration and loci detection highlights the importance of capturing sufficient FnRBCs and ensuring high DNA quality for robust genetic analysis. Observed purity and signal fidelity provide a reliable basis for distinguishing fetal and maternal DNA, supporting the platform’s utility in prenatal diagnostics.

Moreover, the SACA chip’s surface coating enhancements for specificity significantly improve its performance. The application of ε-polylysine (ε-PL) coating increases friction between the chip surface and target cells, reducing the displacement of non-target cells by 31.2%. This enhanced cell retention improves sorting specificity and minimizes contamination. Such surface engineering not only strengthens the platform’s performance but also demonstrates its adaptability for a wide range of biological applications.

Currently, many emerging platforms target trophoblasts for cbNIPD, as these cells are larger and easier to differentiate from the maternal white blood cell background. However, while trophoblasts provide some genetic information, their fragmented state often limits the analysis [15,25]. FnRBCs, on the other hand, represent the true fetal genome and therefore offer superior fidelity for genetic analysis. Trophoblasts and FnRBCs each have distinct advantages and limitations when used as targets for NIPT. Trophoblasts are more abundant in maternal blood and larger in size, making them easier to isolate and process. These factors contribute to the relatively low cost and established nature of trophoblast-targeting platforms [26]. However, trophoblasts often undergo DNA fragmentation and may exhibit placental mosaicism, which can compromise the accuracy of genetic analysis. In contrast, FnRBCs represent the true fetal genome, providing complete and high-quality DNA for detailed genetic analysis [11]. They also have minimal risk of contamination, ensuring reliable results. However, FnRBCs are extremely rare in maternal blood, requiring advanced isolation technologies like the SACA system to efficiently capture them. While this makes the process more technically demanding and costly, FnRBCs offer unparalleled accuracy and reliability for advanced genetic diagnostics. Our SACA system directly addresses this gap, providing a robust method for isolating FnRBCs with minimal contamination and high purity. This capability makes the SACA system a more reliable and accurate platform for cbNIPD compared to conventional cffDNA-based tests, which are prone to confounding factors such as maternal body mass index (BMI), fetoplacental mosaicism, and vanishing twin syndrome [27,28].

Finally, the clinical implications and future directions of this technology are promising. The SACA chip-based platform offers a reliable solution for NIPT by isolating FnRBCs from maternal blood with high purity and precision. Its ability to achieve accurate STR analysis makes it an invaluable tool for fetal genetic screening. Beyond NIPT, this technology has potential applications in other clinical fields, such as isolating circulating tumor cells (CTCs) for cancer diagnostics and monitoring. Continued refinements, particularly in integrating the system into sterile environments, will further expand its clinical utility, bridging the gap between research innovations and practical medical applications [29,30].

## 5. Conclusions

This study establishes the SACA chip as a highly effective and reliable platform for isolating FnRBCs from maternal blood. Its advanced design, precision sorting capabilities, and low detection limits make it a valuable tool for NIPT and other clinical applications. By providing high-quality, contamination-free samples, this technology lays the groundwork for accurate and efficient prenatal diagnostics, ensuring confidence in clinical outcomes and paving the way for future innovations.

## 6. Patents

R.O.C. patent number: TWI825620B.

## Data Availability

The original contributions presented in this study are included in the article. Further inquiries can be directed to the corresponding author.

## Supplementary Materials

The following supporting information can be downloaded at: https://www.mdpi.com/article/10.3390/mi15121515/s1, Figure S1. Workflow for blood sample processing and the SACA system experiment. Figure S2. Principle of the SACA chip. Figure S3. Comparison of the effects of JNC coating and F127 surface modifications on cell capture and retention. Figure S4. White blood cell spike-in experiment using the SACA chip and automated system. Figure S5. False-positive testing using three different antibody combinations in non-pregnant female blood samples. Figure S6. DNA purity and concentration analysis of FnRBC samples obtained from five cases (A–E).

## References

[1] Hurley P.A., Ward R.H., Teisner B., Iles R.K., Lucas M., Grudzinskas J.G. (1993). Serum PAPP-A measurements in first-trimester screening for Down syndrome. Prenat. Diagn..

[2] Falcon O., Auer M., Gerovassili A., Spencer K., Nicolaides K.H. (2006). Screening for trisomy 21 by fetal tricuspid regurgitation, nuchal translucency and maternal serum free beta-hCG and PAPP-A at 11 + 0 to 13 + 6 weeks. Ultrasound Obstet. Gynecol..

[3] Bianchi D.W., Chiu R.W.K. (2018). Sequencing of Circulating Cell-free DNA during Pregnancy. N. Engl. J. Med..

[4] Taylor-Phillips S., Freeman K., Geppert J., Agbebiyi A., Uthman O.A., Madan J., Clarke A., Quenby S., Clarke A. (2016). Accuracy of non-invasive prenatal testing using cell-free DNA for detection of Down, Edwards and Patau syndromes: A systematic review and meta-analysis. BMJ Open.

[5] Bianchi D.W., Flint A.F., Pizzimenti M.F., Knoll J.H., Latt S.A. (1990). Isolation of fetal DNA from nucleated erythrocytes in maternal blood. Proc. Natl. Acad. Sci. USA.

[6] Bianchi D.W., Simpson J.L., Jackson L.G., Elias S., Holzgreve W., Evans M.I., Dukes K.A., Sullivan L.M., Klinger K.W., Bischoff F.Z. (2002). Fetal gender and aneuploidy detection using fetal cells in maternal blood: Analysis of NIFTY I data. National Institute of Child Health and Development Fetal Cell Isolation Study. Prenat. Diagn..

[7] Wang L., Asghar W., Demirci U., Wan Y. (2013). Nanostructured substrates for isolation of circulating tumor cells. Nano Today.

[8] Feng C., He Z., Cai B., Peng J., Song J., Yu X., Sun Y., Yuan J., Zhao X., Zhang Y. (2018). Non-invasive Prenatal Diagnosis of Chromosomal Aneuploidies and Microdeletion Syndrome Using Fetal Nucleated Red Blood Cells Isolated by Nanostructure Microchips. Theranostics.

[9] Hoshino K., Huang Y.Y., Lane N., Huebschman M., Uhr J.W., Frenkel E.P., Zhang X. (2011). Microchip-based immunomagnetic detection of circulating tumor cells. Lab Chip.

[10] Nemescu D., Constantinescu D., Gorduza V., Carauleanu A., Caba L., Navolan D.B. (2020). Comparison between paramagnetic and CD71 magnetic activated cell sorting of fetal nucleated red blood cells from the maternal blood. J. Clin. Lab. Anal..

[11] Wei X., Ao Z., Cheng L., He Z., Huang Q., Cai B., Rao L., Meng Q., Wang Z., Sun Y. (2018). Highly sensitive and rapid isolation of fetal nucleated red blood cells with microbead-based selective sedimentation for non-invasive prenatal diagnostics. Nanotechnology.

[12] Grzywa T.M., Nowis D., Golab J. (2021). The role of CD71+ erythroid cells in the regulation of the immune response. Pharmacol. Ther..

[13] Solomonia N., Playforth K., Reynolds E.W. (2012). Fetal-maternal hemorrhage: A case and literature review. AJP Rep..

[14] Bischoff F.Z., Lewis D.E., Nguyen D.D., Murrell S., Schober W., Scott J., Simpson J.L., Elias S. (1998). Prenatal diagnosis with use of fetal cells isolated from maternal blood: Five-color fluorescent in situ hybridization analysis on flow-sorted cells for chromosomes X, Y, 13, 18, and 21. Am. J. Obstet. Gynecol..

[15] Huang C.-E., Ma G.-C., Jou H.-J., Lin W.-H., Lee D.-J., Lin Y.-S., Ginsberg N.A., Chen H.-F., Chang F.M.-C., Chen M. (2017). Noninvasive prenatal diagnosis of fetal aneuploidy by circulating fetal nucleated red blood cells and extravillous trophoblasts using silicon-based nanostructured microfluidics. Mol. Cytogenet..

[16] Zhu P., Ding J., Zhou J., Dong W.J., Fan C.M., Chen Z.N. (2005). Expression of CD147 on monocytes/macrophages in rheumatoid arthritis: Its potential role in monocyte accumulation and matrix metalloproteinase production. Arthritis Res. Ther..

[17] Yurchenko V., Constant S., Bukrinsky M. (2006). Dealing with the family: CD147 interactions with cyclophilins. Immunology.

[18] Chen T.J., Wu J.K., Chang Y.C., Fu C.Y., Wang T.P., Lin C.Y., Chang H.Y., Chieng C.C., Tzeng C.Y., Tseng F.G. (2014). High-efficiency rare cell identification on a high-density self-assembled cell arrangement chip. Biomicrofluidics.

[19] Chu H.-Y., Lu L.-S., Cho W., Wu S.-Y., Chang Y.-C., Lin C.-P., Yang C.-Y., Lin C.-H., Jiang J.-K., Tseng F.-G. (2019). Enumerating Circulating Tumor Cells with a Self-Assembled Cell Array (SACA) Chip: A Feasibility Study in Patients with Colorectal Cancer. Cancers.

[20] Pratt V.M., Del Tredici A.L., Hachad H., Ji Y., Kalman L.V., Scott S.A., Weck K.E. (2018). Recommendations for Clinical CYP2C19 Genotyping Allele Selection: A Report of the Association for Molecular Pathology. J. Mol. Diagn..

[21] Wu Y., Lu Y.-C., Jacobs M., Pradhan S., Kapse K., Zhao L., Niforatos-Andescavage N., Vezina G., du Plessis A.J., Limperopoulos C. (2020). Association of Prenatal Maternal Psychological Distress With Fetal Brain Growth, Metabolism, and Cortical Maturation. JAMA Netw. Open.

[22] Lu W., Huang T., Wang X.R., Zhou J.H., Yuan H.Z., Yang Y., Huang T.T., Liu D.P., Liu Y.Q. (2020). Next-generation sequencing: A follow-up of 36,913 singleton pregnancies with noninvasive prenatal testing in central China. J. Assist. Reprod. Genet..

[23] Xiang J., Ding Y., Song X., Mao J., Liu M., Liu Y., Huang C., Zhang Q., Wang T. (2020). Clinical Utility of SNP Array Analysis in Prenatal Diagnosis: A Cohort Study of 5000 Pregnancies. Front. Genet..

[24] Pinhas-Hamiel O., Zalel Y., Smith E., Mazkereth R., Aviram A., Lipitz S., Achiron R. (2002). Prenatal diagnosis of sex differentiation disorders: The role of fetal ultrasound. J. Clin. Endocrinol. Metab..

[25] Pfeifer I., Benachi A., Saker A., Bonnefont J.P., Mouawia H., Broncy L., Frydman R., Brival M.L., Lacour B., Dachez R. (2016). Cervical trophoblasts for non-invasive single-cell genotyping and prenatal diagnosis. Placenta.

[26] Vossaert L., Chakchouk I., Zemet R., Van den Veyver I.B. (2021). Overview and recent developments in cell-based noninvasive prenatal testing. Prenat. Diagn..

[27] Jayamohan H., Lambert C.J., Sant H.J., Jafek A., Patel D., Feng H., Beeman M., Mahmood T., Nze U., Gale B.K. (2021). SARS-CoV-2 pandemic: A review of molecular diagnostic tools including sample collection and commercial response with associated advantages and limitations. Anal. Bioanal. Chem..

[28] Hsiao C.H., Chen C.H., Cheng P.J., Shaw S.W., Chu W.C., Chen R.C. (2022). The impact of prenatal screening tests on prenatal diagnosis in Taiwan from 2006 to 2019: A regional cohort study. BMC Pregnancy Childbirth.

[29] Bu X., Zhou S., Li X., Li S., Li H., Ding S., He J., Linpeng S. (2022). A retrospective single-center analysis of prenatal diagnosis and follow-up of 626 chinese patients with positive non-invasive prenatal screening results. Front. Genet..

[30] Li C., Xiong M., Zhan Y., Zhang J., Qiao G., Li J., Yang H. (2023). Clinical Potential of Expanded Noninvasive Prenatal Testing for Detection of Aneuploidies and Microdeletion/Microduplication Syndromes. Mol. Diagn. Ther..

