# Supplementary material for: Automatic Single-Cell Harvesting for Fetal Nucleated Red Blood Cell Isolation on a Self-Assemble Cell Array (SACA) Chip
Source: Micromachines (Basel). 2024 Dec 20;15(12):1515. doi: 10.3390/mi15121515 (PMC11679682; doi:10.3390/mi15121515)
Supplement: Supplementary file 1 [file micromachines-15-01515-s001.zip › micromachines-3373472-supplementary.pdf]

Supplementary

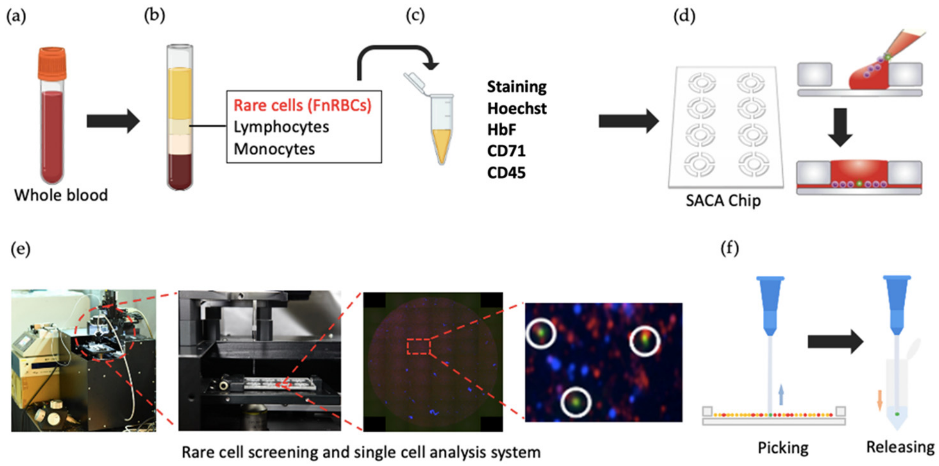

Figure S1. Workflow for blood sample processing and the SACA system experiment. (a) Collection of maternal blood samples. (b) Centrifugation: Peripheral blood mononuclear cells (PBMCs) are isolated using Ficoll centrifugation, followed by two washes with PBS. (c) Staining: Hoechst, antibodies CD71, HbF and CD45 are added to the sample and incubated for 25 minutes, followed by Hoechst staining for an additional 15 minutes. (d) Chip loading: The stained sample is introduced into the SACA chip, allowing cells to settle and self-assemble into a two-dimensional array within 5 minutes. (e) Imaging and cell capture: Using the third-generation imaging system, four spectral band images and a composite image are acquired by scanning the SACA chip. Target cells are identified and isolated using the nested cell capture needle system for single-cell extraction.

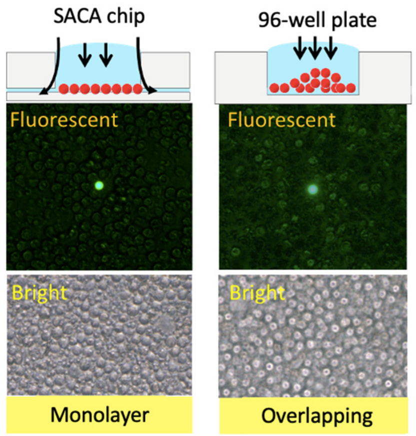

Figure S2. Principle of the SACA chip. Unlike traditional 96-well plates, the SACA chip incorporates evaporation pores that facilitate lateral liquid flow. This unique design enables cells to self-assemble into a two-dimensional array, preventing overlapping and ensuring a uniform distribution.

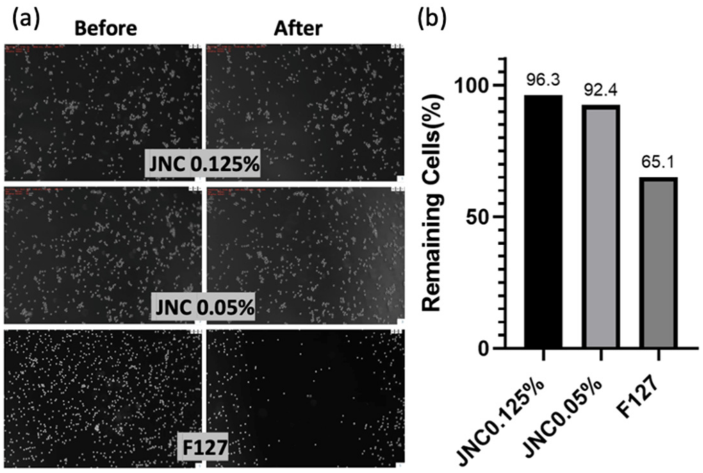

Figure S3. Comparison of the effects of JNC coating and F127 surface modifications on cell capture and retention. (a) Images illustrating cell movement in a fixed region after 30 μL aspiration using a cell needle. Higher concentrations of JNC coating create greater friction between cells, making them less prone to disruption by aspiration forces. This facilitates localized cell extraction, reducing the risk of capturing non-target cells. (b) Quantitative analysis of residual cell counts under different surface modifications, comparing the retention efficiency of JNC and F127 coatings.

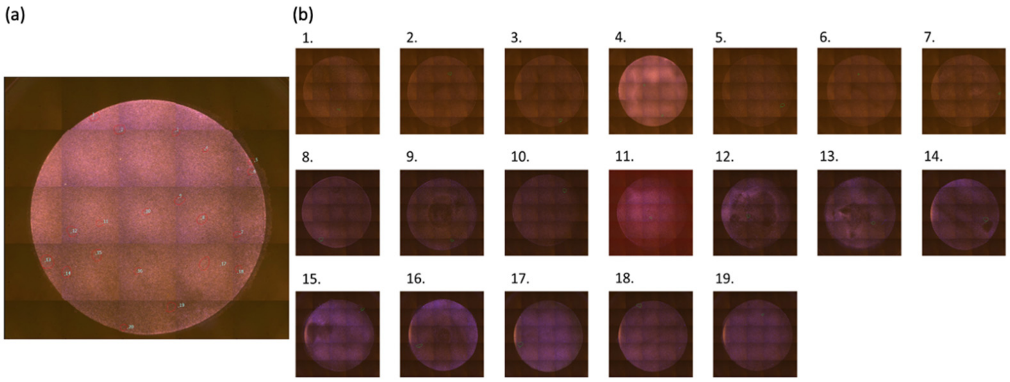

Figure S4. White blood cell spike-in experiment using the SACA chip and automated system. (a) Illustration of the spike-in experiment where 20 FITC-labeled white blood cells were added to a background of 500 million unlabeled cells. (b) Image showing the 20 successfully captured FITC-labeled white blood cells after processing with the SACA chip and automated machine. Notably, the fourth image in (b) highlights a field of view where two target cells are observed, further confirming the system's precision in identifying and isolating multiple cells in a high-background environment.

(a)

	Biomarker combination	HD1	HD2	HD3	Average
A	Hoechst/147/71/HBF/45	5	2.14	4.37	3.83
B	Hoechst/147/71/45	9	5.71	9.37	8.02
C	Hoechst/71/HBF/45	6	3.5	6.2	5.23

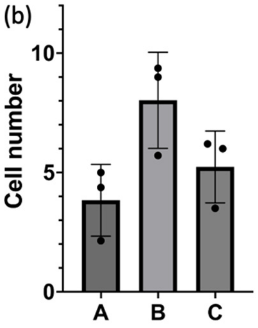

Figure S5. False-positive testing using three different antibody combinations in non-pregnant female blood samples. (a) The number of false-positive cells detected across three healthy donor (HD) samples for each antibody combination. (b) Statistical analysis of the average number of false-positive cells across the three antibody combinations. The standard deviations for the three antibody groups are 1.50, 2.01, and 1.50, respectively, demonstrating variability in specificity among the tested combinations (N=3). Error bars indicate the standard deviation.

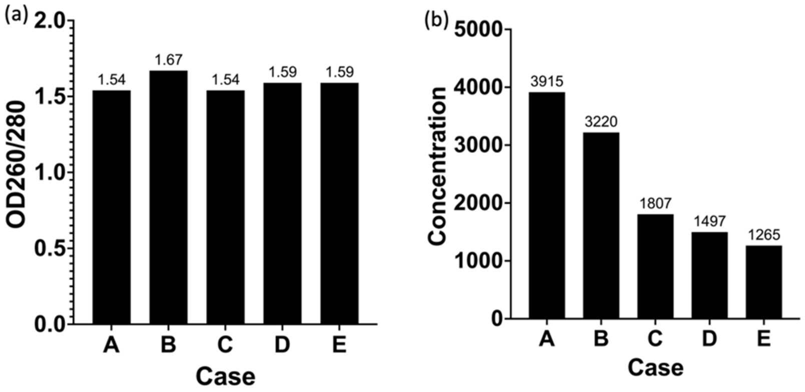

Figure S6. DNA purity and concentration analysis of FnRBC samples obtained from five cases (A–E). (a) OD260/280 ratio of DNA extracted from FnRBC samples across five cases. The ratios range from 1.54 to 1.67, indicating DNA purity slightly below the ideal range of 1.7–1.9 but still acceptable for downstream analysis. (b) DNA concentration of the extracted samples measured in ng/μL. Higher DNA concentrations were observed in cases with more FnRBCs, with values ranging from 1265 ng/μL to 3915 ng/μL, demonstrating a correlation between the number of FnRBCs and DNA yield.
